# Agricultural Fungicide Mancozeb in Leaf Litter Enhances Survival and Growth of *Aedes aegypti* (Diptera: Culicidae)

**DOI:** 10.1002/tox.70056

**Published:** 2026-02-23

**Authors:** Heloise Restello Remus, Gilberto Dinis Cozzer, João Tauchert, Raquel de Brito, Cássia Alves Lima‐Rezende, Jacir Dal Magro, Natalia Bastiani da Luz, Daniel Albeny‐Simões, Renan de Souza Rezende

**Affiliations:** ^1^ Communitarian University of Chapecó Region (Unochapecó) – Chapecó Chapecó Santa Catarina Brazil; ^2^ Ecology Graduate Program Federal University of Pará Belém Pará Brazil

**Keywords:** agrochemical, fitness, mosquito vector, pesticide contamination, trophic interactions

## Abstract

Mancozeb is one of the most widely used agricultural fungicides, exhibiting strong efficacy against a broad spectrum of phytopathogenic fungi. However, its widespread application comes at the cost of environmental contamination. Here, we show the effects of *Inga vera* leaf litter contamination with Mancozeb on life‐history traits of *Aedes aegypti* , focusing on larval mortality, adult wing size, and wing asymmetry. To evaluate the interactive effects of Mancozeb and *I. vera* leaf litter, we tested four treatments prepared from 14‐day leachates of *I. vera* leaves: Control, 0.3, 15, and 40 μg.L^−1^ Mancozeb (*n* = 10 per treatment). Our results revealed that higher Mancozeb concentrations were associated with decreased larval mortality, larger adult body size, and no significant changes in wing asymmetry. These results suggest that the suppression of fungal decomposers and pathogens by Mancozeb preserves organic matter and increases nutrient availability in breeding sites, indirectly enhancing mosquito survival and development. The ecological benefits conferred by Mancozeb on *Ae. aegypti* populations may, in turn, elevate the transmission risk of vector‐borne diseases. By investigating these parameters, we provide insights into how agricultural fungicide contamination in mosquito breeding sites may influence *Ae. aegypti* population dynamics and vector competence in both rural areas and urban centers adjacent to agricultural zones.

## 1 Introduction

The widespread use of agricultural pesticides has raised concerns about their unintended effects on non‐target insect species, which play critical roles in ecosystem functioning [1, 2]. Fungicides and insecticides can cause both acute and chronic toxicity, altering survival, reproduction, and behavior in beneficial insects such as pollinators and natural predators [3, 4]. Studies have shown that neonicotinoids impair foraging efficiency and navigation in bees, reducing colony health and productivity [4, 5]. Pyrethroids and organophosphates disrupt the nervous system of beneficial predators, leading to population declines and reduced biological control services [6]. Additionally, fungicides can interact synergistically with insecticides, exacerbating toxic effects in pollinators and increasing susceptibility to pathogens [7]. The persistence and mobility of these chemicals in agroecosystems further amplify their ecological impact [1, 8], affecting food web dynamics, and biodiversity [9]. Thus, it is crucial to study how the widespread use of agricultural pesticides may influence the ecology, physiology, and evolution of mosquitoes (Diptera: Culicidae), potentially impacting vector control strategies [10].

Insecticides, particularly pyrethroids and organophosphates, exert strong selective pressure on mosquito populations, leading to resistance development through target‐site mutations and enhanced metabolic detoxification [6, 10]. Agrochemical pollutants, including herbicides and fungicides, can also indirectly affect mosquitoes by altering aquatic habitats [11], modifying microbial communities [12], and influencing larval development and survival [13]. Studies have shown that exposure to sublethal concentrations of pesticides can accelerate resistance evolution, enhance detoxification enzyme activity, and even increase tolerance to insecticides used in public health programs [10, 11]. Additionally, agrochemical contamination in breeding sites can shape mosquito life‐history traits, such as sex ratio and body size, which may influence their vectorial capacity [14]. It is crucial to explore the integration of agricultural and vector control practices to better understand how pesticide exposure influences non‐target mosquitoes, such as *Aedes* (*Stegomyia*) *aegypti* (Linnaeus, 1762) (Diptera: Culicidae), and its implications for the efficacy of control measures in rural areas and urban zones surrounded by agriculture [10].

Although *Ae. aegypti* is predominantly associated with urban environments and artificial container habitats, increasing evidence shows that its occurrence is not restricted to densely populated city centers [15]. In many tropical and subtropical regions, *Ae. aegypti* persists in peri‐urban and rural landscapes, where it exploits natural and semi‐natural breeding sites, particularly in areas surrounding agricultural activities [15, 16]. Reports of oviposition in plant axils, tree holes, and small water‐holding structures in rural environments indicate that the species can interact with detritus‐based microhabitats under certain conditions [17, 18]. These transitional zones between agricultural fields and human dwellings represent potential interfaces for exposure to fungicide‐contaminated runoff, especially during rainfall events, irrigation flows, and leaching from crop residues [19].

Agricultural fungicides may significantly affect Culicidae mosquitoes, as *Ae. aegypti*, by altering their development, survival, and susceptibility to insecticides [20]. Exposure to fungicides like Mancozeb and Azoxystrobin has been shown to delay larval development, increase pupal mortality, and reduce adult emergence rates in culicids [10, 20]. Mancozeb belongs to the ethylene‐bis‐dithiocarbamate (EBDC) class of pesticides and is a dithiocarbamate fungicide containing manganese (Mn) and zinc (Zn) in its formulation [21]. It is one of the most used fungicides in agricultural practices worldwide [22, 23]. Fungicides may also interfere with metabolic detoxification pathways, leading to increased vulnerability to pathogens and potential interactions with insecticide resistance mechanisms [20, 23]. Additionally, sublethal concentrations of fungicides may induce physiological stress, affecting reproductive success and population dynamics [10, 20, 22].

In natural aquatic systems, Mancozeb has been detected at concentrations ranging from 0.3 to 39.2 μg/L [24, 25]. Given the widespread use and environmental persistence of agricultural fungicides such as Mancozeb, it is crucial to assess their ecological impact on vector mosquito populations [21, 22], particularly *Ae. aegypti*, and the potential implications for disease transmission in both rural areas and urban centers adjacent to agricultural zones [26, 27]. Despite the well‐documented ecological effects of Mancozeb, no studies have yet explored its impact on the life history traits of *Ae. aegypti*. Understanding these interactions is essential to elucidate the ecological implications of pesticide contamination in both rural areas and urban centers near agricultural zones.

In this context, the widespread use of Mancozeb in agriculture raises concerns about its potential effects on *Ae. aegypti* populations, due to its persistence in the environment and capacity to contaminate aquatic mosquito breeding sites [20]. This study aims to evaluate the effects of Mancozeb‐treated leaf litter, representing a common vector of fungicide entry into aquatic systems, on key life‐history traits of *Ae. aegypti*, including larval mortality, adult wing size, and wing asymmetry. We used senescent leaves to simulate the natural input of organic matter into the aquatic system and its subsequent leaching in the presence of Mancozeb. Even after the fungicide is no longer present in the water, its effects on the leaf material persist, potentially due to alterations in the organic matter cycling process (decomposition), which may modify the quality and availability of resources for aquatic organisms. By investigating these parameters, we may provide insights into how agricultural fungicide contamination in mosquito breeding sites would influence *Ae. aegypti* population dynamics and vector competence in both rural areas and urban centers near agricultural zones.

This study is based on the assumptions that: (i) exposure to Mancozeb disrupts the development of various aquatic organisms by inducing oxidative stress, developmental delays, and increased mortality [21]; and (ii) *Ae*. *aegypti* larvae develop in water bodies that may be contaminated with agrochemicals in both rural areas and urban centers near agricultural zones of subtropical regions [28, 29]. Based on these premises, we hypothesize that exposure to sublethal and lethal concentrations of agricultural pesticides (as Mancozeb fungicide) will negatively affect mosquito life‐history traits. Specifically, we predict that: (i) larval mortality will increase with rising concentrations of Mancozeb applied to the leaf litter because the fungicide is expected to alter litter‐associated microbial communities and diminish the nutritional quality of the detritus, reducing the energy available for larval development due to toxicity and metabolic stress resulting from resource‐mediated exposure; (ii) adult wing size will decrease as a consequence of developmental stress and resource allocation trade‐offs during metamorphosis, driven by the consumption of fungicide‐contaminated detritus; and (iii) wing asymmetry will increase, reflecting developmental instability caused by environmental stressors associated with ingestion of Mancozeb‐altered leaf material during larval growth.

## 2 Material and Methods

### 2.1 Mosquito Source and Experiment Site

The *Ae. aegypti* mosquito larvae used in this experiment were obtained from a laboratory‐maintained colony at the Laboratory of Ecological Entomology (LABENT‐Eco), housed at the Communitarian University of the Chapecó Region (Unochapecó). The founding population of this colony originated from eggs provided by Chapecó's Epidemiological Surveillance, and wild strains are annually introduced to preserve genetic variability, although no strict generational control is implemented (for more see also [30, 31, 32]).

The insectary operates under controlled conditions, maintaining a temperature of 27°C ± 2°C, relative humidity of 70%–80%, and a 12:12‐h light–dark photoperiod. Larvae are reared on a *Spirulina*‐based fish food diet at a concentration of 0.15 g/L, administered every 3 days to ensure adequate nutritional intake for colony maintenance [33]. Adult mosquitoes are provided with a 20% sugar solution, replenished every 3 days, and receive weekly blood meals to sustain reproductive activity.

For the experiment, oviposition strips were submerged in 1 L of distilled water to induce egg hatching. After 24 h, larval density was assessed, yielding ~500–600 first‐instar larvae per beaker. Newly hatched larvae (< 24 h old) were carefully counted and transferred into experimental microcosms. The experiment was conducted under identical environmental conditions to those used in colony maintenance, minimizing potential stress‐induced variability in larval development. To isolate the effect of the fungicide on leaf litter, we maintained minimal resource concentrations (Spirulina Alcon at 0.15 g/L) in the studied system.

### 2.2 Leaf Litter Collection and Mancozeb Concentrations

To collect senescent leaves of *Inga vera* Willd., 1 m^2^ nets were suspended 1.5 m above the ground beneath the selected tree. All collected leaves originated from the same individual tree and were obtained during a single collection event. The collection site coordinates were 27° 05′ 34.7″ S and 52° 39′ 57.6″ W.

Following collection, leaves were air‐dried for 72 h, after which 15 g of dried leaves were immersed in 2 L of distilled water within a Beaker flask and allowed to leach for 14 days. A control treatment consisting solely of distilled water was established (0 μg/L), alongside three Mancozeb‐treated conditions, featuring low (0.3 μg/L), medium (15 μg/L), and high (40 μg/L) concentrations of the fungicide (*n* = 10 each; Figure 1). After 14 days, the leaf litter was oven‐dried at 40°C for 48 h and subsequently ground using a Shimadzu AUW220D grinder.

**FIGURE 1:**
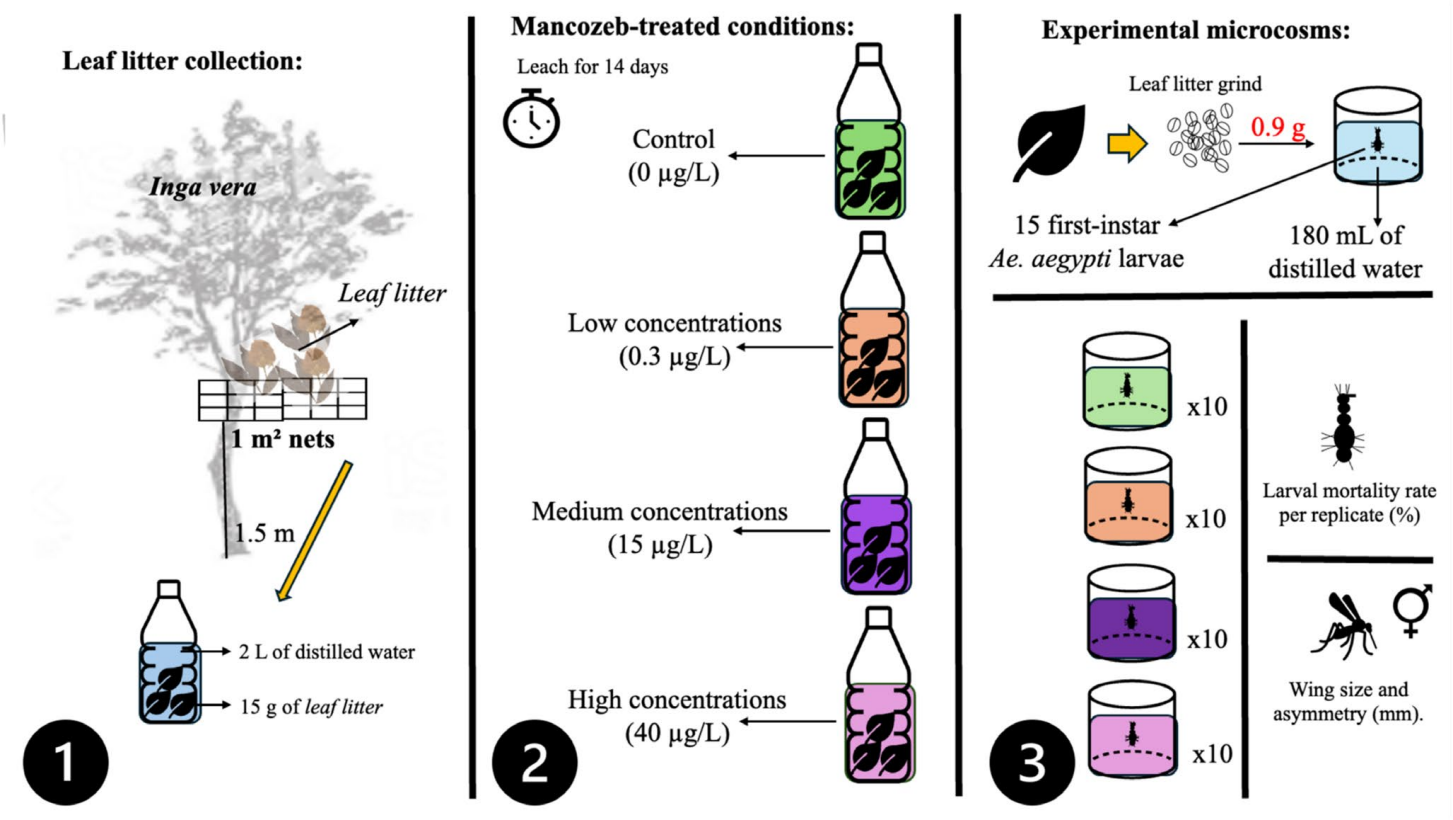
Schematic summary of microcosm experiments to investigate the Mancozeb effects on life history of the *Aedes aegypti* mosquito.

### 2.3 Chemical Characterization of Leaves Litter

The chemical composition of the bioactive fraction in *I. vera* leaf leachates at the beginning of the experiment was analyzed by Gas Chromatography–Mass Spectrometry (GC–MS) using the FullScan method. The chemical composition of the bioactive fraction of *I. vera* leaf leachates was analyzed exclusively for the control treatment (without Mancozeb addition), with the objective of characterizing the baseline chemical profile of senescent leaves under standardized leaching conditions. No GC–MS analyses were conducted on Mancozeb‐treated leachates, and therefore no direct chemical comparisons among treatments were performed. This characterization was intended to support ecological interpretation by identifying naturally occurring compounds with potential biological activity, rather than to quantify treatment‐induced chemical changes. For the analysis, 200× reductions of the leachate were concentrated from solid‐phase extraction with SPE cartridges, where 5 mL eluted with methanol from the control leachate treatment were transferred to a 2.5 mL vial for injection into the chromatograph. GC–MS analysis was performed using the HS‐trap method. The GC–MS analysis was conducted using the HS‐trap method on an Agilent 7890B gas chromatograph coupled to a 5977A quadrupole mass spectrometer (Agilent Technologies, Palo Alto, CA, USA). Helium was used as the carrier gas at a flow rate of 1.2 mL min^−1^. Sample injection was performed using the CTC‐PAL sampler (Agilent Technologies). The HS‐trap oven temperature was set to 70°C for 10 min, with helium as the carrier gas at a column head pressure of 150 kPa.

The GC temperature program began at 40°C (held for 2 min), then increased to 200°C at 5°C/min, followed by a ramp to 300°C at 40°C/min (held for 3 min). The MS transfer line temperature was maintained at 250°C, while the ion source temperature was set to 230°C. Electron ionization (EI) was used for compound detection, operating at 70 eV with a mass scan range of m/z 40–400. Analyte identification was performed by comparing the mass spectra against the National Institute of Standards and Technology (NIST 5.01) library, accessed through the Agilent P/N G1033A control system.

### 2.4 Experimental Microcosms and Procedures

Each microcosm was designed as an entomological trap and consisted of a plastic container divided into two sections (Figure 1). The lower section was filled with a 0.9 g aliquot of the processed litter suspended in 180 mL of distilled water (according to the assigned treatment) plus 0.15 g/L of *Spirulina* as a food source, a minimum possible resource for mosquitoes, following Cozzer et al. (2023). For food preparation, *Spirulina* was weighed using an analytical balance (Bel Engenharia SKU M—0.0001 g) and mixed in distilled water for 3 min to ensure homogeneity. A total of 15 first‐instar *Ae. aegypti* larvae were introduced into each experimental microcosm (*n* = 10 per concentration; Figure 1). Microcosms were checked daily to record the number of dead *Ae. aegypti* larvae, pupae, and adults. Emerging adults, captured in the upper section of the microcosm, were anesthetized using carbon dioxide (CO_2_), placed into labeled Eppendorf tubes according to treatment and replicate, and subsequently killed and stored at −18°C for further analysis.

For each dead adult, sex was determined, and both wings were measured based on the ventral view, as a proxy for allometric variation [34, 35]. Wing measurements were conducted using a Zeiss Stemi 305 binocular stereoscopic microscope at ×40 magnification. All images were calibrated to maintain consistent magnification, ensuring the wing was centered in the visual field to enable accurate size comparisons and minimize optical distortion. In each experimental replicate, several variables were assessed, including: (i) Larval mortality rate per replicate (%); (ii) Wing size (mm) (average of both wings used as a proxy for body size) [34]; and (iii) Wing asymmetry (mm). Wing size asymmetry was obtained as the absolute difference in length of the two wings, divided by mean wing length for the corresponding individual to avoid a positive variance to mean ratio. All measurements were taken individually for each sample to ensure precision in analyzing morphological variation.

### 2.5 Statistical Analysis

A one‐way generalized linear mixed‐effect model (GLMM) was implemented using the glmer function from the lme4 package [36] to assess the effect of leaf litter treated with different concentrations of Mancozeb (0, 0.3, 15, and 40 μg.L^−1^) on the larval mortality rate, adult size, and wing asymmetry of *Ae. aegypti* [37]. For larval mortality rate, the GLMM included each experimental container (replicate) as a random effect to account for within‐group variance. For adult wing size and wing asymmetry, mosquito sex (male or female) and microcosm identity (container) were incorporated as random effects to control for variation associated with individual containers and potential sex‐based differences in morphological traits.

The error distribution of all models was evaluated using the hnp function [38] to check for overdispersion or underdispersion, ensuring model accuracy. The best‐fitting error distribution was Gaussian for larval mortality rate and wing size and Poisson for wing asymmetry. Following the GLMM analysis, a Tukey post hoc test was conducted to perform pairwise comparisons between treatments for each dependent variable. Additionally, a one‐way generalized linear model (GLM) was separately applied to adult wing size and wing asymmetry, analyzing each sex independently due to the presence of sexual dimorphism, which could influence statistical outcomes. All analyses were conducted using R software (version 4.1.3; R Core Team, 2014).

## 3 Results

### 3.1 Effects of Mancozeb on *Ae. aegypti* Life History Traits

We found a significant effect of Mancozeb concentration on decreasing the larval mortality rate of *Ae. aegypti* (χ^2^ = 24.7, *p* < 0.001; Table 1a; Figure 2a). The highest mortality was recorded in the Control (73% ± 16.91%), followed by Low (53% ± 26.17%), High (10% ± 9.81%), and Mid (6% ± 6.57%) concentrations (Figure 2a). Specifically, we found significant pairwise differences between Control and Mid concentrations (Tukey tests; *p* < 0.001), Control and High concentrations (Tukey tests; *p* = 0.001), and Low and Mid (Tukey tests; *p* = 0.040) concentrations (Table 2a).

**TABLE 1:** One‐way factorial generalized linear mixed effects (GLMME; a, b, and c) and one‐way generalized linear models (GLM, d, e, and f) were performed to compare the effect of treatments on life history traits of *Ae. aegypti* mosquito. Larval mortality rate (a) considered replicates as a random effect, and wing size (b) and wing asymmetry (c) considered sex and replicates as random effects.

	df	logLik/sum sq	deviance/Mean Sq	Chi‐sq/F–values	*p*
GLMME					
a. Larval mortality rate					
Null model	3	−9.85	19.71		
Treatments	6	2.50	−5.01	24.71	< 0.001
b. Wing size					
Null model	4	−180.75	361.51		
Treatments	7	−166.65	333.30	28.21	< 0.001
c. Wing asymmetry					
Null model	4	412.83	−825.66		
Treatments	7	413.72	−827.44	1.78	0.619
GLM					
d. Wing size in male					
Treatment	3	2.43	0.81	6.91	< 0.001
e. Wing size in female					
Treatment	3	1.88	0.63	4.26	0.006
f. Wing asymmetry in male					
Treatment	3	0.1	0.03	0.54	0.658
g. Wing asymmetry in female					
Treatment	3	0.13	0.04	0.89	0.447

**FIGURE 2:**
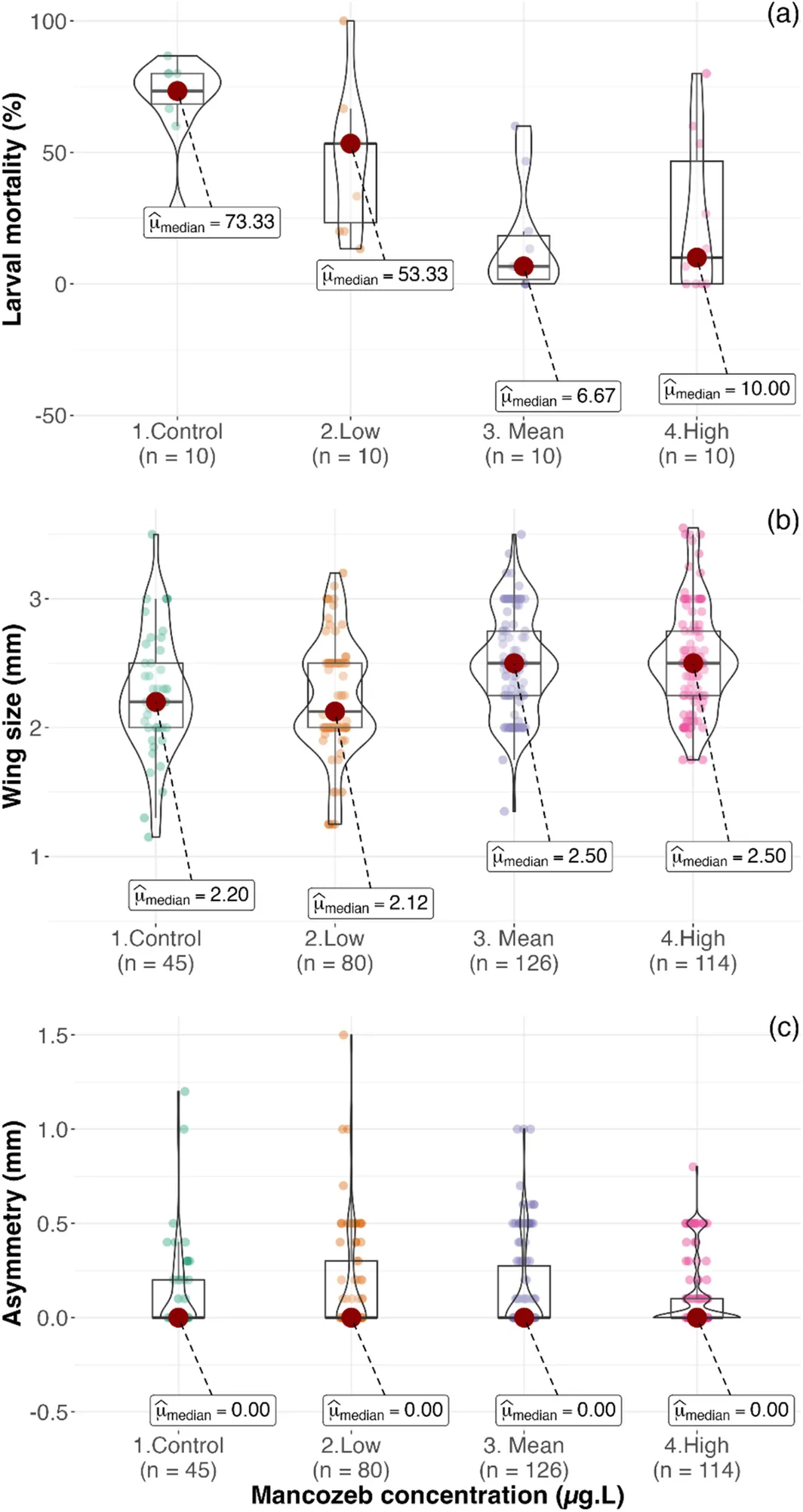
Larval mortality (in %; a), and wing size (in mm; b) and wing asymmetry (in mm; c) of different Mancozeb concentration treatments (Control, Low, Mid, and High). The violin boxes represent the quartiles; the brown circles in the horizontal represent the average; the horizontal‐bold line represents the median; and the vertical line represents the upper and lower limits.

**TABLE 2:** Tukey post hoc results showed statistical differences between Mancozeb distinct concentrations (control, low, mid, and high) in larval mortality rate (a), and wing size (b). Pairwise comparison (contrast), Standard Error (SE), degrees of freedom (df), and *p* values (*p*).

Contrast	Estimate	SE	df	t.ratio	*p*
a. Larval mortality rate					
Control–low	0.235	0.107	27	2.19	0.151
Control–mid	0.539	0.107	27	5.03	< 0.001
Control–high	0.460	0.107	27	4.29	0.001
Low–mid	0.304	0.107	27	2.84	0.04
Low–high	0.225	0.107	27	2.10	0.179
Mid–high	−0.079	0.107	27	−0.74	0.881
b. Wing size					
Control–low	−0.008	0.099	31	−0.08	0.999
Control–mid	−0.227	0.096	27	−2.38	0.105
Control–high	−0.268	0.097	28	−2.76	0.046
Low–mid	−0.219	0.052	333	−4.19	< 0.001
Low–high	−0.259	0.056	343	−4.64	< 0.001
Mid–high	−0.040	0.049	340	−0.82	0.844

For mosquito wing size, we also found a significant effect of the treatment (χ^2^ = 28.2, *p* < 0.001; Table 1b; Figure 2b). The smallest mosquitoes were observed in the Low concentration treatment (2.1 ± 0.44 mm), followed by the Control (2.2 ± 0.47 mm), High (2.5 ± 0.4 mm), and Mid (2.5 ± 0.38 mm) concentrations (Figure 2b). We found significant pairwise differences between Control and High concentrations (Tukey tests; *p* < 0.046), Low and Mid concentrations (Tukey tests; *p* < 0.001), and Low and High concentrations (Tukey tests; *p* < 0.001; Table 2b). Regarding wing asymmetry, Mancozeb concentration did not significantly affect this trait (χ^2^ = 1.7, *p* = 0.619, Table 1c). The mean wing asymmetry values were 0.0017 (± 0.0028 mm) for the Low concentration, 0.0015 (± 0.0025 mm) for the Control, 0.001 (± 0.0024 mm) for the Mid concentration, and 0.001 (± 0.0019 mm) for the High concentration (Figure 2c).

To assess sex‐specific effects, separate analyses were performed for males and females (Table 1d–f; Figure 3). In males (Figure 3a), Mancozeb concentration positively significantly influenced wing size (F = 6.9, *p* < 0.001), with Tukey's post hoc tests showing differences between Low and Mid concentrations (Tukey tests; *p* < 0.001) and Low and High concentrations (Tukey tests; *p* < 0.001). Similarly, in females (Figure 3b), Mancozeb concentration also had a significant effect on wing size (F = 4.2, *p* = 0.006) with significant pairwise comparisons between the Low and High concentrations (Tukey tests; *p* < 0.001) and between the Control and High concentrations (Tukey tests; *p* < 0.001). In contrast, wing asymmetry was not significantly affected by Mancozeb concentration in either males (F = 0.5, *p* = 0.658; Table 1e; Figure 3c) or females (F = 0.8, *p* = 0.447; Table 1f; Figure 3d).

**FIGURE 3:**
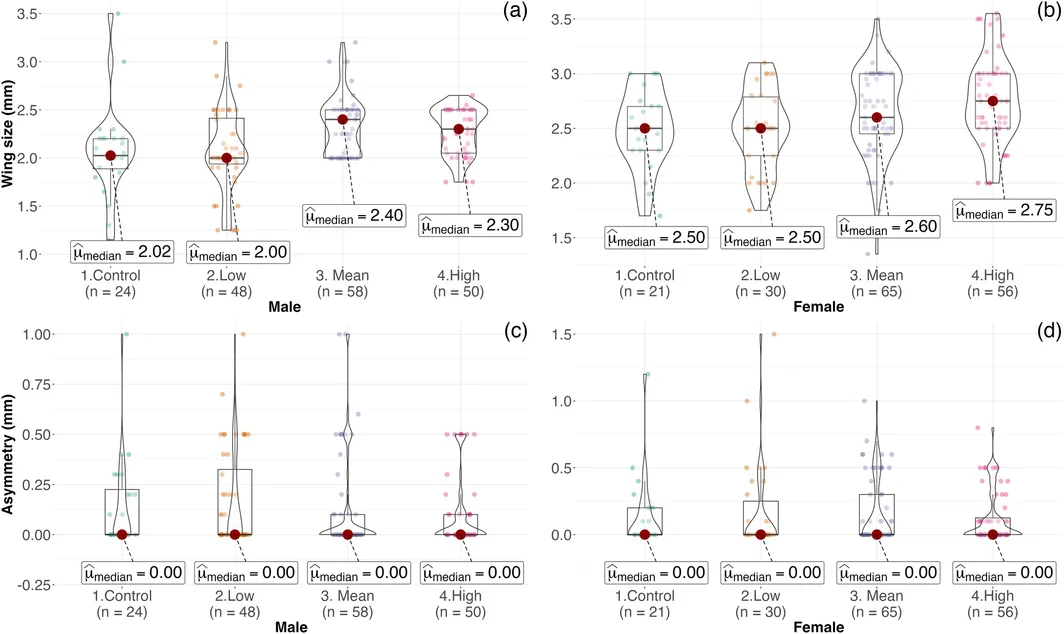
Responses in wing size (a and b; in mm), and wing asymmetry (c and d; in mm) of different sexes (males and females) to different Mancozeb concentration treatments (Control, Low, Mid, and High). The violin boxes represent the quartiles; the brown circles in the horizontal represent the average; the horizontal‐bold line represents the median; and the vertical line represents the upper and lower limits.

### 3.2 Leachates Chemical Analysis

Chemical analysis of *Inga vera* leachates identified seven volatile compounds that simultaneously met the criteria for spectral confirmation (NIST 5.01) and chromatographic consistency, exhibiting ≤ ±100 units of difference between the experimental retention index and the calculated Kovats indices (Table 3). These compounds constitute the final reliable chemical profile of the leachates and represent the only constituents suitable for ecological interpretation in this study (Table 3). The chromatogram revealed a predominance of terpenoid, phenolic, steroidal, and lactonic molecules, collectively accounting for 2.07%–30.98% of the total detected signal, with retention times ranging from 6.46 to 14.57 min. The dominant compound was α‐Santalol (30.98%), a sesquiterpene alcohol. Two acetylated tetradecenol esters (7‐methyl‐Z‐ and 10‐methyl‐8‐tetradecen‐1‐ol acetates) were also detected, along with low‐abundance constituents such as 2,4‐bis(1,1‐dimethylethyl)phenol, belonging to the phenolic class, and 1,4‐bis(trimethylsilyl)benzene, categorized as an organosilane. The leachate profile additionally included a sesquiterpene ketone (Spiro[4.5]decan‐7‐one) and a γ‐butyrolactone derivative (7,9‐di‐tert‐butyl‐1‐oxaspiro(4,5)deca‐6,9‐diene‐2,8‐dione).

**TABLE 3:** Relative area (%) and chemical classification of compounds identified in leachate from *Inga vera* leaves by GC–MS, including retention.

Compost	Percent compound (%)	Retention value	Kovats	Compost class
α‐santalol	30.98	1454	1495	Terpenes
Spiro[4.5]decan‐7‐one, 1,8‐dimethyl‐8,9‐epoxy‐4‐isopropyl—	29.97	1626	1727	Sesquiterpene ketone
7,9‐di‐tert‐butyl‐1‐oxaspiro(4,5)deca‐6,9‐diene‐2,8‐dione	19.96	1929	1943	Lactona
10‐methyl‐8‐tetradecen‐1‐ol acetate	7.23	1822	1709	Acetates
Phenol, 2,4‐bis(1,1‐dimethylethyl)—	6.20	1555	1520	Phenol
7‐methyl‐Z‐tetradecen‐1‐ol acetate	3.60	1822	1709	Acetates
1,4‐bis(trimethylsilyl)benzene	2.07	1124	1070	Organosilicon monomers

## 4 Discussion

### 4.1 Larval Mortality

Our findings revealed a counterintuitive pattern, whereby higher Mancozeb concentrations in leaf litter microcosms reduced larval mortality (from 73% in the control to 6% at mid‐concentrations) and produced larger *Ae. aegypti* adults. Mancozeb is a broad‐spectrum fungicide widely used to prevent fungal colonization on agricultural surfaces such as leaves, flowers, and fruits [21]. Upon contact with water, Mancozeb may degrade into secondary toxic compounds, releasing high concentrations of manganese and zinc [23]. These metals may promote the formation of reactive oxygen species, inducing oxidative stress at the cellular level, leading to structural damage, replication errors, and apoptosis [39, 40]. Such toxicity is typically linked to increased mortality rates in exposed organisms [23, 41]. However, our findings reveal an inverse relationship between Mancozeb concentration and *Ae. aegypti* larval mortality, suggesting a possible alternative mechanism influencing survival. Unlike most previous studies that primarily evaluated the direct effects of pesticide exposure, our approach focused on the indirect effects mediated by the availability of resources that had undergone leaching in the presence of the fungicide.

A possible ecological explanation is that the leaching process in the presence of the fungicide may affect both decomposer fungi and pathogenic microorganisms associated with the leaf material. Although we did not directly measure fungal abundance, diversity, or decomposition rates in our experiment, previous studies have shown that the suppression of fungal decomposers [42] and pathogens [12], may reduce the microbial breakdown of organic matter [43], thereby increasing the availability of undecomposed resources for other organisms. This proposed mechanism should therefore be interpreted as plausible but still requiring direct empirical confirmation. Ultimately, this higher resource availability may create a more favorable condition for mosquito larvae [33, 44], even in scenarios of increased larval density. This positive effect on mosquito life‐history surprisingly persists despite increased competition due to higher larval densities resulting from lower mortality rates in these treatments [32, 45]. These results may suggest that Mancozeb use in mosquito breeding habitats could inadvertently promote higher vector populations, underscoring the need for integrated pest management strategies that account for such ecological interactions in both rural areas and urban centers adjacent to agricultural zones. This also highlights the necessity for pesticide regulations to incorporate comprehensive risk assessments that consider sublethal and indirect effects on non‐target insects.

More specifically, a possible explanation is that Mancozeb's fungicidal activity may alter the microbial composition of *Ae. aegypti* larval habitats [12, 46]. Aquatic fungi, such as the class Hyphomycetes, play a critical role in organic matter decomposition [42, 47, 48] and may function as natural pathogens (as *Metarhizium brunneum*) of mosquito larvae [49], contributing to larval mortality. By suppressing these fungal communities, Mancozeb may slow leaf litter decomposition, preserving organic material and increasing nutrient availability in breeding sites [50, 51, 52]. This shift in microbial dynamics could create a more stable and nutrient‐enriched environment [43], ultimately favoring larval development and enhancing survival rates [32, 46]. Additionally, sublethal exposure to Mancozeb may induce physiological stress responses in mosquito larvae, potentially triggering hormesis effects, where low doses stimulate adaptive mechanisms that improve survival [20, 53].

The chemical analysis of *Inga vera* leaf leachates identified 16 compounds, including α‐Santalol and Phenol, 2,4‐bis(1,1‐dimethylethyl)‐, all of which have known antioxidant and cytoprotective properties. Although part of these bioactive compounds was removed during the leaching process, it is likely that they were not completely lost from the leaf material. The persistence of antioxidant and cytoprotective molecules in the decomposing detritus could mitigate oxidative stress in mosquito larvae, offering protective effects during development. In addition, the presence of Mancozeb may further enhance the quality of the detritus by reducing fungal decomposition, thereby preserving nutrient quantity and quality. Together, these factors likely created more favorable conditions for *Ae. aegypti* larvae, ultimately leading to improved survival and greater individuals, despite the initial exposure to fungicide‐contaminated leaf material. To specifically assess the effect of the fungicide on leaf litter decomposition, we maintained minimal external nutrient supplementation (from *Spirulina*) in the studied system [33]. These findings highlight the importance of considering both chemical and ecological interactions when evaluating the impact of pesticides on mosquito populations, particularly in rural and peri‐urban areas adjacent to agricultural landscapes.

As our experiment was conducted under controlled laboratory conditions using a single leaf litter species ( *I. vera* ), generalization to natural environments should be made cautiously. *Ae. aegypti* populations in the field are exposed to heterogeneous detrital pools, variable physicochemical conditions, and more diverse microbial communities, which may alter the strength or direction of the observed responses [16, 28]. In addition, leaf species differ in chemical composition and interactions with fungicides, which could influence larval development and resource quality [32, 54]. Although the concentrations used were environmentally relevant, the relatively large intervals between treatments may have limited the detection of threshold or non‐linear dose–response patterns. Future studies using finer concentration gradients and longer exposure periods, ideally across multiple mosquito generations, would help clarify these dynamics.

Mancozeb is widely used in small‐scale and industrial agriculture, and residues are frequently detected in surface waters, drainage channels, and temporary ground pools near crop‐producing communities [19, 55]. In rural and peri‐urban settlements, where dengue, Zika, and Chikungunya are increasingly recorded, the overlap between agricultural fungicide use, human habitation, and mosquito breeding sites may foster indirect exposure pathways [56]. While such exposure is likely of limited relevance in high‐density urban centers, this agrochemical‐vector interface may be ecologically meaningful in rural and agricultural regions, where vector‐borne disease transmission remains significant [11, 56]. Therefore, the use of *Ae. aegypti* in this study provides insight into how fungicide contamination could influence mosquito fitness in settings where agricultural practices and arbovirus transmission converge. We also acknowledge that additional field‐based evidence on exposure routes would further strengthen this ecological contextualization, and future research specifically addressing these transitional habitats is warranted.

### 4.2 Adult Size and Asymmetry

Adults emerging from higher Mancozeb contamination levels during leaf litter leaching exhibited increased body sizes. This outcome may be attributed to the suppression of pathogenic fungi [12] and the enhanced availability of food resources derived from leaf litter [32, 57], both of which could facilitate mosquito development. Typically, microcosms with higher mortality rates yield larger *Ae. aegypti* adults due to reduced intraspecific competition and the decomposition of dead larvae, which serves as an additional nutrient source [30, 45]. Developing insect larvae allocate a substantial portion of assimilated energy toward structural growth, meaning that increased food availability often results in shorter development times and larger adult body sizes [53, 58]. As a consequence of improved nutritional conditions, emerging adults also tend to exhibit greater body symmetry [45].

An interesting and somewhat unexpected finding was that mosquitoes did not exhibit significant wing asymmetry values in the Mancozeb treatments. Insects developing in stable, resource‐rich environments tend to show higher developmental stability, resulting in larger and more symmetrical individuals [30, 33]. Conversely, environmental stressors that disrupt growth often lead to increased asymmetry in bilaterally symmetrical organisms [59, 60, 61]. Although it is also possible that fluctuating asymmetry is not a sufficiently sensitive biomarker of sublethal Mancozeb stress for *Ae. aegypti*. The use of additional physiological or immunological metrics may provide better resolution of subtle stress responses in future work. The observed differences in body size between males and females align with the natural sexual dimorphism of *Ae. aegypti* [35], where females consistently achieve larger sizes due to their greater reproductive investment [58].

On the other hand, Mancozeb's indirect effects on trophic dynamics may further promote *Ae. aegypti* populations by disrupting ecological interactions that regulate mosquito survival and adult body size. By reducing fungal communities responsible for organic matter decomposition [42, 47, 48], Mancozeb may preserve plant litter and increase nutrient availability in aquatic breeding sites [33], indirectly facilitating mosquito proliferation. This altered microbial environment likely enhances food resource availability for larvae [43], potentially contributing to higher survival rates and the emergence of larger adult mosquitoes. Additionally, the suppression of fungal pathogens that naturally infect *Ae. aegypti* larvae could further elevate population densities [10, 12, 46]. The ecological benefits conferred by Mancozeb on *Ae. aegypti* life history traits and population dynamics may, in turn, heighten the risk of vector‐borne disease transmission, including dengue, Zika, and chikungunya, thereby exacerbating public health challenges. These findings highlight the urgent need for integrated pest and agricultural management strategies that consider the indirect ecological consequences of fungicide use on mosquito vectors and their broader implications for disease transmission in both rural areas and urban centers adjacent to agricultural zones.

## 5 Conclusion

Our findings highlight the complex ecological effects of Mancozeb on *Ae. aegypti* populations, demonstrating that its fungicidal activity can indirectly enhance mosquito survival and development by altering microbial communities and nutrient dynamics in aquatic habitats. By suppressing fungal decomposers and pathogens, Mancozeb preserves organic matter and increases resource availability, leading to higher larval survival, larger adult body sizes, and greater developmental stability, refuting our hypothesis. This proposed mechanism remains inferential in the absence of direct measurements of fungal dynamics and decomposition and should be interpreted with caution. These physiological advantages may ultimately contribute to increased vector populations, exacerbating the risk of disease transmission. The unintended consequences of agricultural fungicide use underscore the need for an integrated pest management approach that considers both direct and indirect ecological impacts on vector species in both rural areas and urban centers adjacent to agricultural zones. Because our experiment was carried out under controlled laboratory conditions, using a single leaf species ( *I. vera* ) and relatively broad concentration intervals, the generalization of these responses to natural settings requires careful consideration. Further research incorporating multiple litter substrates, finer concentration gradients, and longer‐term exposure, ideally across generations and under field‐realistic microbial and environmental conditions, is needed to determine the ecological relevance and consistency of these effects.

Despite these limitations, our results raise important implications for vector ecology and public health, particularly in agricultural and peri‐urban environments where *Ae. aegypti* may coexist with fungicide runoff. If confirmed under natural conditions, such indirect positive effects of fungicides could influence mosquito population dynamics and, consequently, arbovirus transmission potential. We therefore emphasize the need to integrate agrochemical contamination into discussions of mosquito ecology, vector management, and One Health approaches.

## Author Contributions

R.S.R. conceived the study. H.R.R., G.D.C., J.T., and R.B. collected field data. R.S.R. managed and analyzed the data. H.R.R. wrote the manuscript with feedback from R.S.R., G.D.C., J.T., R.B., D.A.‐S., J.D.M., N.B.L., and C.A.L.‐R.

## Funding

This work is supported by the National Council for Scientific and Technological Development (CNPq) (grant 302044/2022‐1), the Foundation for the Support of Research and Innovation of the State of Santa Catarina (FAPESC) (grants 2023TR001470, 2023TR001511, 2024TR2016, 2024TR2015, 2024TR002336, and 2024TR2013).

## Ethics Statement

The experimental procedures were conducted following ethical guidelines for invertebrate use in research.

## Consent

The authors have nothing to report.

## Conflicts of Interest

The authors declare no conflicts of interest.

## Data Availability

The data that support the findings of this study are available from the corresponding author upon reasonable request.
